# Root litter decomposition is suppressed in species mixtures and in the presence of living roots

**DOI:** 10.1111/1365-2745.14207

**Published:** 2023-10-13

**Authors:** Cristina Heredia‐Acuña, Marina Semchenko, Franciska T. De Vries

**Affiliations:** ^1^ Department of Microbiology Royal College of Surgeons in Ireland Dublin 2 Ireland; ^2^ Department of Earth and Environmental Sciences University of Manchester Manchester UK; ^3^ Institute of Ecology and Earth Sciences University of Tartu Tartu Estonia; ^4^ Institute for Biodiversity and Ecosystem Dynamics University of Amsterdam Amsterdam The Netherlands

**Keywords:** biotic interactions, home‐field advantage, litter mixture, plant diversity, rhizosphere priming effect, root exudates, root litter decomposition

## Abstract

1. Plant species diversity and identity can significantly modify litter decomposition, but the underlying mechanisms remain elusive, particularly for root litter. Here, we aimed to disentangle the mechanisms by which plant species diversity alters root litter decomposition. We hypothesised that (1) interactions between species in mixed communities result in litter that decomposes faster than litter produced in monocultures; (2) litter decomposition is accelerated in the presence of living plants, especially when the litter and living plant identities are matched (known as home‐field advantage).
2. Monocultures and a mixture of four common grassland species were established to obtain individual litter and a ‘natural’ root litter mixture. An ‘artificial’ mixed litter was created using litter from monocultures, mixed in the same proportions as the species composition in the natural litter mixtures based on qPCR measurements. These six root litter types were incubated in four monocultures, a four‐species mixture and an unplanted soil.
3. Root decomposition was strongly affected by root litter identity and the presence, but not diversity, of living roots. Mixed‐species litter decomposed slower than expected based on the decomposition of single‐species litters. In addition, the presence of living roots suppressed decomposition independent of the match between litter and living plant identities. Decomposition was not significantly different between the ‘natural’ and ‘artificial’ root litter mixtures, indicating that root‐root interactions in species mixtures did not affect root chemical quality.
4. *Synthesis*. Suppressed decomposition in the presence of living roots indicates that interactions between microbial communities associated with living roots and root litter control root litter decomposition. As we found no support for the importance of home‐field advantage or interspecific root interactions in modifying decomposition, suppressed decomposition of mixed‐species litter seems to be primarily driven by chemical rather than biotic interactions.

## Graphical abstract

Suppressed decomposition in the presence of living roots indicates that interactions between microbial communities associated with living roots and root litter control root litter decomposition. As the authors found no support for the importance of home‐field advantage or interspecific root interactions in modifying decomposition, suppressed decomposition of mixed‐species litter seems to be primarily driven by chemical rather than biotic interactions.

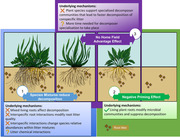

## 1 INTRODUCTION

The loss of plant species diversity represents a serious risk to many ecosystems and can significantly undermine ecosystem functioning, including soil carbon storage (Hautier et al., 2018; Loreau et al., 2001; Prommer et al., 2019). The decomposition of dead plant tissues (litter) is a key component of the soil carbon (C) cycle. The role of living plants, their identity and diversity, in litter decomposition processes is still poorly understood, particularly for root litter. Although roots represent 50%–90% of total biomass production and form 70% of soil C inputs in grasslands (Birouste et al., 2012; Freschet et al., 2013), only a small number of studies have focused on root litter decomposition (Man et al., 2020; Poirier et al., 2018; See et al., 2019; Silver & Miya, 2001; Zhang et al., 2008; Zhang & Wang, 2015). Even less is known about how root litters belonging to different species decompose in mixtures (Prieto et al., 2017). Furthermore, few studies have investigated the multiple pathways by which biotic interactions can modify decomposition (Huangfu et al., 2019; Yang et al., 2021).

The rate of litter decomposition is influenced by litter chemical properties (Poirier et al., 2018), soil abiotic conditions (Fanin et al., 2020) as well as complex biotic interactions (Barel et al., 2019; Joly et al., 2017; Veen et al., 2019). The effects of litter diversity on decomposition have been primarily studied using leaf litter. Positive effects have been attributed to nutrient transfer from high‐quality to low‐quality litter within the mixture, microenvironmental effects, niche complementarity among decomposers and the optimisation of resource acquisition across soil trophic groups (Hättenschwiler & Gasser, 2005; Lin et al., 2021; Trogisch et al., 2016). Negative effects of litter mixing on decomposition have been attributed to microbial immobilisation and inhibitory secondary compounds (Chomel et al., 2016; Hättenschwiler et al., 2005). Root litter decomposition rates have been shown to be correlated with chemical (i.e. C:N ratio; Man et al., 2020) and morphological traits (i.e. root diameter, specific root length; Liu et al., 2019). For instance, Liu et al. (2019) found that litters with lower root diameters decomposed faster. Other studies have found that higher N concentrations and lower C:N ratios did not increase fine root decomposition (Dong et al., 2016; Sun et al., 2018, but see Chen et al., 2017). Root diameter and specific root length have been found to correlate with fungal community composition, while root chemical traits such as phenol content, influenced bacterial diversity (Bitao et al., 2022), suggesting that root traits control root microbial decomposers (Sun et al., 2018). Man et al. (2020) showed that the presence of root litter with high C:N reduced decomposition through decreasing microbial biomass, suggesting that litter‐microbe interactions control decomposition (Man et al., 2020; Prieto et al., 2017).

Both chemical and morphological root traits can be modified when plants grow together. Plant roots can detect the proximity and identity of other individuals, both at the level of genotype and species, and respond to neighbour diversity by modifying growth and root traits (i.e. specific root length; Dudley & File, 2007; Peng & Chen, 2021). Interactions with genetically distant genotypes or other species have been shown to enhance root production (Bartelheimer et al., 2006), and increase specific root length and root N content (Bartelheimer et al., 2006; Bu et al., 2017; Semchenko et al., 2014; Wambsganss et al., 2021). Thus, such changes in root traits through biotic interactions have the potential to modify root litter decomposition, but are rarely considered (Semchenko et al., 2017).

Besides the role of litter properties, root litter decomposition can be significantly modified by the presence of living plants (Barel et al., 2019). This phenomenon is known as the rhizosphere priming effect. It can be either positive, with living roots enhancing the decomposition of organic matter via the release of root exudates, or negative if living roots suppress decomposition (Kaštovská et al., 2015; Yin et al., 2021; Zheng et al., 2023). The latter can be driven by the exudation of inhibitory molecules or by competition between the decomposer community and roots (Guenet et al., 2010; Saar et al., 2016) or root‐associated mycorrhizal fungi (Gadgil & Gadgil, 1975). Furthermore, the diversity of living plants has been shown to either slow (Chen et al., 2017; Joly et al., 2017) or enhance litter decomposition via changes in microenvironmental conditions (Wambsganss et al., 2021). Finally, plant litter can decompose faster near the plant where it originates from compared to another location (known as home‐field advantage; Ayres et al., 2009). Evidence suggests that home‐field advantage is tightly linked with specialised decomposer communities. For example, Veen et al. (2019) found that after 12 months of incubating leaf litter, a unique microbiome developed. In this study, the abundance of a particular fungal taxon, *Sordariomycetes*, was the main driver of home‐field advantage. Similarly, other studies found that the positive or negative rhizosphere priming effect on litter decomposition was determined by the presence or absence of specific bacterial species (Ayres et al., 2009; Gholz et al., 2000; Huangfu et al., 2019; Vivanco & Austin, 2008). On the other hand, home‐field disadvantage has been linked to litter quality and environmental factors (Veen et al., 2018). Litter decomposition can therefore be modified by living plant communities via multiple mechanisms and their relative importance remains unknown.

Here, we independently manipulated the composition of root litter and living plant community to disentangle how plant species monocultures and mixtures differ in root litter decomposition. In addition, we determined the importance of priming and home‐field advantage effects on litter decomposition in plant species monocultures and mixtures. We hypothesised that (i) the decomposition of mixed‐species root litter is faster than predicted from the decomposition of single species litters due to enhanced resource use complementarity among decomposers exposed to chemically diverse litter, (ii) natural litter mixtures (i.e. root litter mixtures produced by plants grown in species mixtures) will decompose faster than artificial mixtures (i.e. root litter mixtures prepared from plants grown in species monocultures) as interspecific root interactions have been shown to result in higher quality litter (i.e. lower C:N ratios) and (iii) root litter will decompose faster when incubated under plant communities compared to unplanted soil because of the positive rhizosphere priming effect, and especially in the presence of conspecific plants because of a positive home‐field advantage.

To test these hypotheses, we performed a greenhouse experiment that consisted of two phases. In the first phase, we grew monocultures and a four‐species mixture of four common grassland species to create root litter. We determined the relative abundance of each species in the mixtures using a quantitative real‐time polymerase chain reaction method (Mommer et al., 2008). Based on this information, we constructed litter mixtures from roots grown in monocultures. In the second phase, we incubated the six litter types (artificial mixture, natural mixture, and the four monoculture litters) under plants grown in monoculture, a four‐species mixture or bare soil.

## 2 MATERIALS AND METHODS

### 2.1 Production and preparation of root litter bags (Phase 1)

We used four common grassland species to produce root litters: two grasses (*Anthoxanthum odoratum* L. and *Dactylis glomerata* L.) and two forbs *(Leucanthemum vulgare* Lam. and *Rumex acetosa* L.). The species were chosen to represent different growth forms and growth strategies (slow‐growing *A. odoratum* and *L. vulgare*, and fast‐growing *D. glomerata* and *R. acetosa*; Grime et al., 1988). Plants were grown as species monocultures (48 replicates per species) or four‐species mixtures (1:1:1:1; 48 replicates). We created 48 individual monoculture root litters, 48 root litter mixtures from plants that were grown together (‘natural’ mixtures) and 48 root litter mixtures that we constructed using monoculture litters (‘artificial’ mixtures). We grew a total of 240 pots and treated every pot as a separate replicate because mixing between samples might generate inaccurate or invalid results (Reinhart & Rinella, 2016). Therefore, each litter used in the final experiment was produced in a separate experimental unit (or four separate experimental units in the case of ‘artificial’ mixtures). The communities were sown in pots of 3.5 L pots filled with commercial sandy loam soil (NH_4_^+^‐N: 4.41 μg g^−1^ dry soil, NO_3_‐N: 4.25 μg g^−1^ dry soil, total nitrogen: 9.08 mg g^−1^ dry soil). They were kept under greenhouse conditions (6–20°C and 16/8 h day/night cycle) with a soil moisture level of 60% water holding capacity (WHC). Root litter harvest was staggered over the course of a week after 6 months of growth to prevent degradation of roots used for the qPCR analysis (see next section). Root litter harvest was done by cutting plant biomass at soil surface level, after which root systems were rinsed to remove soil and gently pressed in absorbent paper to take away excess water. Special care was taken in this step to avoid variation once the samples were weighed. Subsamples of all mixtures and monocultures were taken and immediately frozen for later use in the qPCR analysis to determine species contributions to root biomass (see the next section). The remaining root biomass was then dried at 60°C until constant weight. Litter length was standardised by hand‐cutting to 1 cm fragments (Liu et al., 2006). We made litter bags using a 1 mm polyester mesh, measuring 5 × 5 cm: we added a total of 0.1 g of dried root litter per bag.

### 2.2 Quantification of relative species proportions in root biomass of natural mixtures

The relative contribution of individual species to mixed‐community root biomass was assessed by qualitative real‐time polymerase chain reactions (qPCR). We use the following primers for *A. odoratum* (FW 5′‐TCATGTACTGTTGTACTGCGAAG‐3′, RV 5′‐GAATCAAGCTGGACAGTAAATGAC‐3′), *D. glomerata* (FW 5′‐CAGGGCATTGAACTGATGATG‐3′, RV 5′‐AGAAACTGGTGTGCGTCTGC‐3′), *L. vulgare* (FW 5′‐AAACTCTACAGGCGTTCTTCC‐3′, RV 5′‐ATTTCACTTCATAGCTCTTCACTG‐3′) and *R. acetosa* (FW 5′‐CCTCGCCGTCTTCAAGAACT‐3′, RV 5′‐CTCAGTTGCCGTTTTGTCCA‐3′) reported by Mommer et al. (2018) and Wagemaker et al. (2021) for performing the qPCR following the protocol described by Mommer et al. (2008). Briefly, a set of standards with an equal proportion of roots per species (1:1:1:1) and ratio mixtures were made using individual species' roots from monoculture treatments. Proportions used for these ratio mixtures were 6:3:1:0, giving six combinations per species and thus 24 ratio mixtures (4 species × 6 ratio combinations × 3 replicates; Figure S1). Root tissue DNA extraction was done using the DNeasy® (QIAGEN©, Germany) plant kit and later quantified using a NanoDrop 2000® spectrophotometer (Thermo Scientific Inc). The following protocol was used for qPCR reactions: we combined 10 μL of the master mix DyNAmo Flash SYBR Green qPCR Kit® (ThermoFisher Scientific), 0.75 μL of MgCl_2_, 2 μL of genomic DNA (1 ng/μL), 0.6 μL of primer (4 μM for each) and 12.65 μL of miliQ water, for a final reaction volume of 20 μL. The reaction was run in the StepOnePlus™ Real‐Time PCR System 96 well (ThermoFisher Scientific) using the following parameters: initial denaturation for 10 min at 95°C, denaturation through 40 cycles of 15 s at 95°C, 45 s at 62°C and a standard melting curve (Mommer et al., 2010). We then used the information on relative species' contributions of 13 ‘natural’ mixtures to construct 48 litter bags for the artificial mixtures. Although all species mixtures were composed of four species, species relative abundances varied between artificial litter mixtures (4%–53% for *A. odoratum*, 6%–56% for *D. glomerata*, 3%–51% for L. vulgare and 5%–33% for *R. acetosa*) mimicking variation in natural mixtures based on qPCR results. We also prepared 48 litterbags from each species' monoculture and 48 natural mixture litterbags for incubation in the next phase of the experiment.

### 2.3 Experimental design of the main experiment (Phase 2)

**FIGURE 1:**
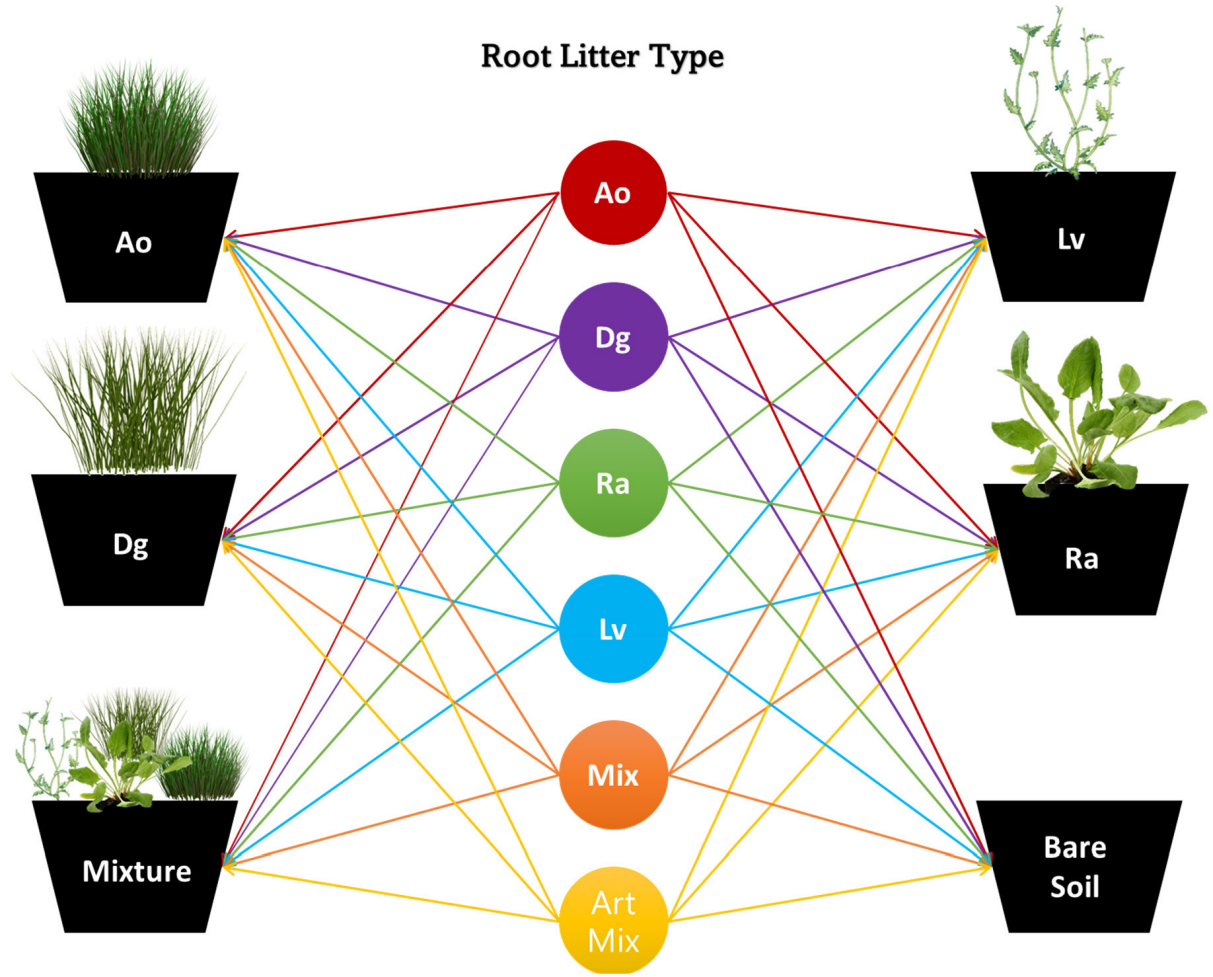
Set‐up for the second phase of the litter decomposition experiment. Six litter types (denoted with coloured circles, produced by four species monocultures or a four‐species mixture and an artificial mixture composed of monoculture litters) were incubated in five plant communities (denoted with black trapezoids, four species monocultures and a four‐species mixture) or unplanted, bare soil. Ao, *Anthoxanthum odoratum*; ArtMix, artificial mixture; Bare Soil, bare soil; Dg, *Dactylis glomerata*; Lv, *Leucanthemum vulgare*; Mix, natural mixture; Ra, *Rumex acetosa*.

Soil was collected in June 2016 at Colt Park (54°11′37″ N, 2°20′55″ W), which is part of the Ingleborough National Nature Reserve (NNR) in the Yorkshire Dales, UK. The hay meadow is traditionally managed and is classified as an MG3, *Anthoxanthum odoratum*–*Geranium sylvaticum* grassland (Rodwell, 1992). All species used in our study were present in the area. The soil had a pH_H2O_ of 6.18, a total N of 0.57 ± 0.02%, and a total C of 5.7 ± 0.14%, and was classified as clayey brown earth soil. The soil was sieved (4 mm) and homogenised. We established four monocultures (*Anthoxanthum odoratum*, *Leucanthemum vulgare*, *Rumex acetosa*, *Dactylis glomerata*), and one mixed community, alongside a bare soil treatment. All communities consisting of four individuals, in 1 L (10 cm height, 13 cm diameter) pots filled with homogenised soil (640 ± 0.01 g; Figure 1). The plant communities were left to grow for 3 months with constant soil moisture (60% WHC) at 16–20°C and 16/8 h day/night light cycle. After the communities grew for 3 months, two litterbags of a single treatment were buried in each pot with the top of the bag located at 5 cm depth (288 litterbags in total). Each plant community received litterbags of each litter type (Figure 1). Thus, the experiment had 36 treatments, with four replicates per treatment, resulting in 144 pots arranged in a randomised block design. The litter bags were positioned vertically in the middle of each pot.

The first and second litter bags from each pot were retrieved after 2 and 4 months, respectively. Litter bags were cleaned of soil and ingrown roots. Afterward, they were dried at 60°C for 48 h. The proportion of litter mass remaining was calculated by dividing the final dry weight by the initial dry weight of the litter.

### 2.4 Soil nutrient analyses

At the final harvest, when the second set of litter bags was collected, the soil was sieved (2 mm) and homogenised. Dissolved organic nitrogen (DON) and dissolved organic carbon (DOC) were extracted by shaking soil samples (5 g of soil in 35 mL of milli‐Q water) on an orbital shaker (HS 501; IKA® Germany) at 250 rev min^−1^ for 10 min. Then, they were centrifuged at 4560 rev min^−1^ for 30 min and filtered through a Whatman No. 1 filter paper (GE Healthcare Life Sciences, USA; Forster, 1995). Plant‐available inorganic N (NO_3_‐N and NH_4_‐N) was extracted using 0.5 M KCl (25 mL per 5 g of soil). All samples were shaken on a horizontal shaker at 250 rev min^−1^ for 30 min, centrifuged at 4560 rev min^−1^ for 30 min and filtered through a Whatman no. 1 filter (GE Healthcare Life Sciences, USA). Both extracts were measured on an auto‐analyser AA3 (SEAL®, UK) by colorimetry. Dissolved organic carbon concentration was measured by combustion oxidation on a TOC‐L analyser (Shimadzu, Japan).

Microbial biomass C and N were determined using the chloroform‐fumigation‐extraction (CFE) method (Wu et al., 1990). Five grams of soil were fumigated with ethanol‐free chloroform under vacuum for 24 h at room temperature. After the incubation, the chloroform was removed by evaporation. Fumigated and unfumigated samples were shaken in 25 mL of 0.5 M K_2_SO_4_ on an orbital shaker (HS 501; IKA® Germany) at 250 rev min^−1^ for 30 min. Subsequently, the samples were centrifuged at 4560 rev. min^−1^ for 30 min and filtered through a Whatman No. 1 (GE Healthcare Life Sciences, USA). The extract was transferred into a glass tube and diluted 1:4 with mili‐Q water and organic C concentration in fumigated and non‐fumigated extracts was analysed with an auto‐analyser AA3 (colorimetry; SEAL®, UK). Microbial biomass N was analysed by measuring the total N concentration in fumigated and unfumigated extracts on an auto‐analyser AA3 (colorimetry; SEAL®, UK). Microbial biomass C and N were calculated using the difference between fumigated and unfumigated samples, using a *K*_EN_ of 0.54 (Brookes et al., 1985) and *K*_EC_ of 0.45 (Joergensen et al., 2011; Vance et al., 1987) as correction factors.

### 2.5 Root litter chemical analysis

A total of 144 samples (48 monocultures, 48 natural mixtures and 48 artificial mixtures) of root litter were ground to a fine powder with a mill‐ball (MM400; Restch GmbH©), and 5 mg were encapsulated in tins (D1008; Elemental Analysensysteme GmbH©) to measure total C and N on an elemental analyser (Vario EL cube; Elementar Analysensysteme GmbH©).

### 2.6 Microbial community analysis

Soil microbial community composition was analysed using phospholipid fatty acid (PLFA) which allows the assaying of biomass and composition of microbial communities. However, it is important to consider that presence/absence of specific groups or species diversity cannot be determined (Frostegård et al., 2011). We followed the Buyer and Sasser (2012) protocol. Briefly, 0.5 g of freeze‐dried soil was transferred in test tubes with Bligh‐Dyer extractant containing an internal standard (1,2‐dinonadecanoyl‐sn‐glycero‐3‐phosphocholine; Avanti Polar Lipids, USA). Lipid separation was performed using a solid phase extraction plate (SPE) and eluted with 0.5 mL of 5:5:1 methanol: chloroform: MiliQ water, after which the samples were centrifuged to dry in a vacuum centrifuge (miVac®, GENEVAC LTD, UK). Finally, the samples were dissolved in transesterification reagent (0.2 mL, 0.134 g KOH; 18 mL methanol; 6 mL toluene) and incubated for 15 min at 37°C. After the incubation, 0.075 M acetic acid and chloroform were added, and samples were vortexed for 30 s. The bottom layer (chloroform phase) was transferred into a clean glass vial. All the chloroform was evaporated in a vacuum centrifuge just to dryness at room temperature. Finally, the samples were dissolved in 75 μL of hexane. Afterward, the samples were transferred to small‐volume glass inset GC vials. Fatty acid methyl esters (FAMEs) were separated using a gas chromatograph (Agilent Technologies, 7890B, GC Systems, USA). The PLFAs 15:0, i15:0, i16:0, 16:0ω9, i17.0, cy17:0, 18:1ω7 and cy19:0 were chosen to represent bacterial PLFAs and 18:2ω9 and 18:1ω9 were indicators of fungal PLFAs. The ratio of 18:2ω6 to bacterial PLFAs was taken to represent the ratio of fungi to bacteria in soil. Gram‐positive bacteria were represented by the branched fatty acids: i15, a15, i16, and i17, while Gram‐negative bacteria were represented by cy17, cy19 and 18:1ω7 (Bardgett et al., 1996, 2001).

### 2.7 Data analysis and calculations

All analyses were performed using R software (version 3.6.3; R Core Team, 2020). Litter decomposition rates were analysed with linear mixed models (LMM) using the packages car (Fox & Weisbery, 2019), lme4 (Bates et al., 2013) and emmeans (Lenth, 2022). The proportion of litter mass remaining undecomposed was used as the response variable. Litter type (four monocultures, natural and artificial mixtures), plant community identity (four monocultures, mixture, bare soil), time (two sampling points) and their interactions were used as fixed factors. Differences in the decomposition of monoculture versus natural mixture litter in the presence of monoculture versus mixed plant communities were analysed with a LMM. Using litter type and community type coded as monoculture or mixture. Differences in decomposition between artificial and natural mixtures were analysed using a LMM with litter mixture type (natural or artificial) as a fixed factor. The expected decomposition of mixed litter was calculated as the sum of the litter mass remaining of individual species monocultures multiplied by the individual species' proportions in the mixture based on qPCR. The difference between the observed and expected decomposition was calculated and its deviation from zero analysed using a *t*‐test (lm function, intercept only model). An LMM model was performed with the difference between observed and expected decomposition as the response variable and plant community as a fixed factor. To test if the difference between observed and expected decomposition depended on the plant community that the litter was placed in. Priming effects were assessed using a LMM with plant community type (planted or bare soil), litter type and their interaction as fixed factors. To test if differences in decomposition between litter types were more distinct in bare or planted soil. Two separate models were performed using data from bare soil and planted soil, and the proportion of variance explained by litter type was compared using marginal *R* ^2^ from package *performance* (Lüdecke et al., 2021). Home‐field advantage was analysed using an LMM with litter match to the plant community (‘home’ or ‘away’), litter type and their interaction as fixed factors. Only data on monoculture plant communities and monoculture litter were used to test home‐field advantage. Since two litterbags were inserted in each pot and harvested at two sampling points, all models on litter decomposition included time and interactions with other predictors as fixed factors and block and pot nested in a block as random factors. The influence of plant community and litter type, and their interaction, on soil nutrients, microbial biomass and composition was analysed using an LMM, with experimental block as a random factor. Model residuals were normally distributed and variances did not significantly differ between groups (Levene test, *p* > 0.05). Hence, decomposition data did not require transformation to satisfy model assumptions. Pairwise differences between means were tested with Tukey's HSD test.

## 3 RESULTS

### 3.1 Effects of litter and plant community identity on litter decomposition rates

**FIGURE 2:**
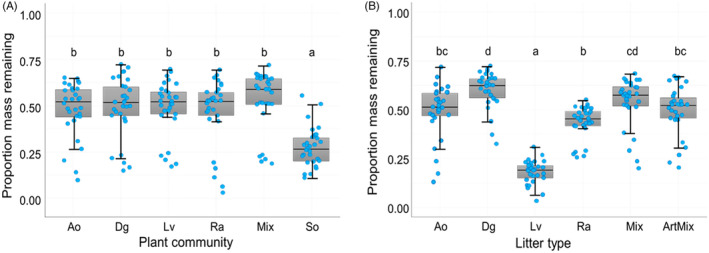
Root litter decomposition as affected by plant community (A) and litter type (B). Boxplots represent litter decomposition data for two sampling points pooled together (central lines are medians, box top and bottom are first and third quartiles, whiskers are 1.5 times the interquartile range, blue triangles are raw data points). Different letters indicate a statistically significant difference between groups (*p* < 0.05, Tukey HSD). Ao, *Anthoxanthum odoratum*; ArtMix, artificial mixture; Bare, bare soil; Dg, *Dactylis glomerata*; Lv, *Leucanthemum vulgare*; Mix, natural mixture; Ra, *Rumex acetosa*.

Litter decomposition was significantly affected both by root litter type (*F*_5,105_ = 64.33, *p* < 0.001) and plant community identity (*F*_5,105_ = 25.05, *p* < 0.001). Decomposition rates were significantly higher in unplanted soil than in any of the planted communities, while there was no significant difference in decomposition between planted communities (Figure 2A). *L. vulgare* root litter had the fastest decomposition rates of all root litter types (*p <* 0.001, Tukey test), followed by *R. acetosa* and *A. odoratum* (Figure 2B). *D. glomerata* litter had the slowest decomposition rates, and both natural and artificial mixtures had intermediate rates. No significant interactive effects between litter type and plant community were detected (*F*_25,105_ = 1.45, *p* = 0.100).

### 3.2 Decomposition of root litter produced in species monocultures, mixtures or artificially mixed litter

**FIGURE 3:**
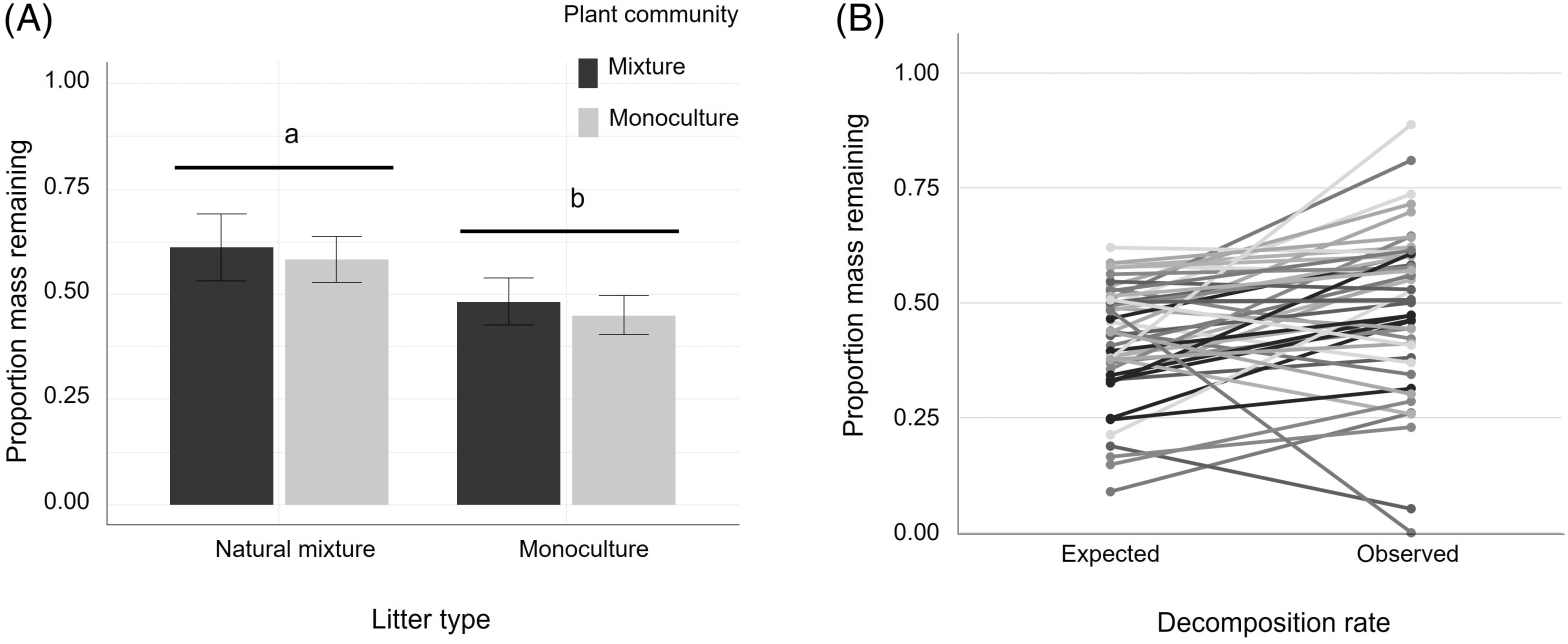
(A) Decomposition of root litter produced by species monocultures or natural mixtures when placed in monoculture (grey) or mixed plant communities (black). Bars show the mean model estimates and their standard error. Different letters indicate a statistically significant difference between groups (*p* < 0.05). (B) Expected and observed decomposition rates for each artificial root litter mixture. There was a statistically significant difference between expected and observed decomposition (*p* < 0.05).

The decomposition of litter originating from mixed communities was significantly slower than mean decomposition of litter originating from monocultures (*F*_1,93_ = 9.65, *p* = 0.003) but litter decomposition was not significantly affected by the identity of plant community where the litter was placed (Figure 3A; Table S1). There were no significant differences in the decomposition rates of natural and artificial litter mixtures (*F*_1,33_ = 2.62, *p* = 0.115; Table S1). Observed decomposition rates of the artificial litter mixtures were significantly lower than expected based on the decomposition of monoculture litters (*p* = 0.002, *t*‐test; Figure 3B). The identity of the live plant community did not significantly modify the difference between observed and expected rates of decomposition of artificial litter mixtures (*F*_5,15_ = 2.04, *p* = 0.131).

Similarly, decomposition of natural litter mixtures was also significantly slower than expected based on the decomposition of monoculture litters (*p* < 0.001, *t*‐test, Figure S2). The deviation from expected decomposition was not significantly correlated with the relative abundances of different species in the litter mixture (Figure S3).

### 3.3 The effect of plant presence on litter decomposition rates

**FIGURE 4:**
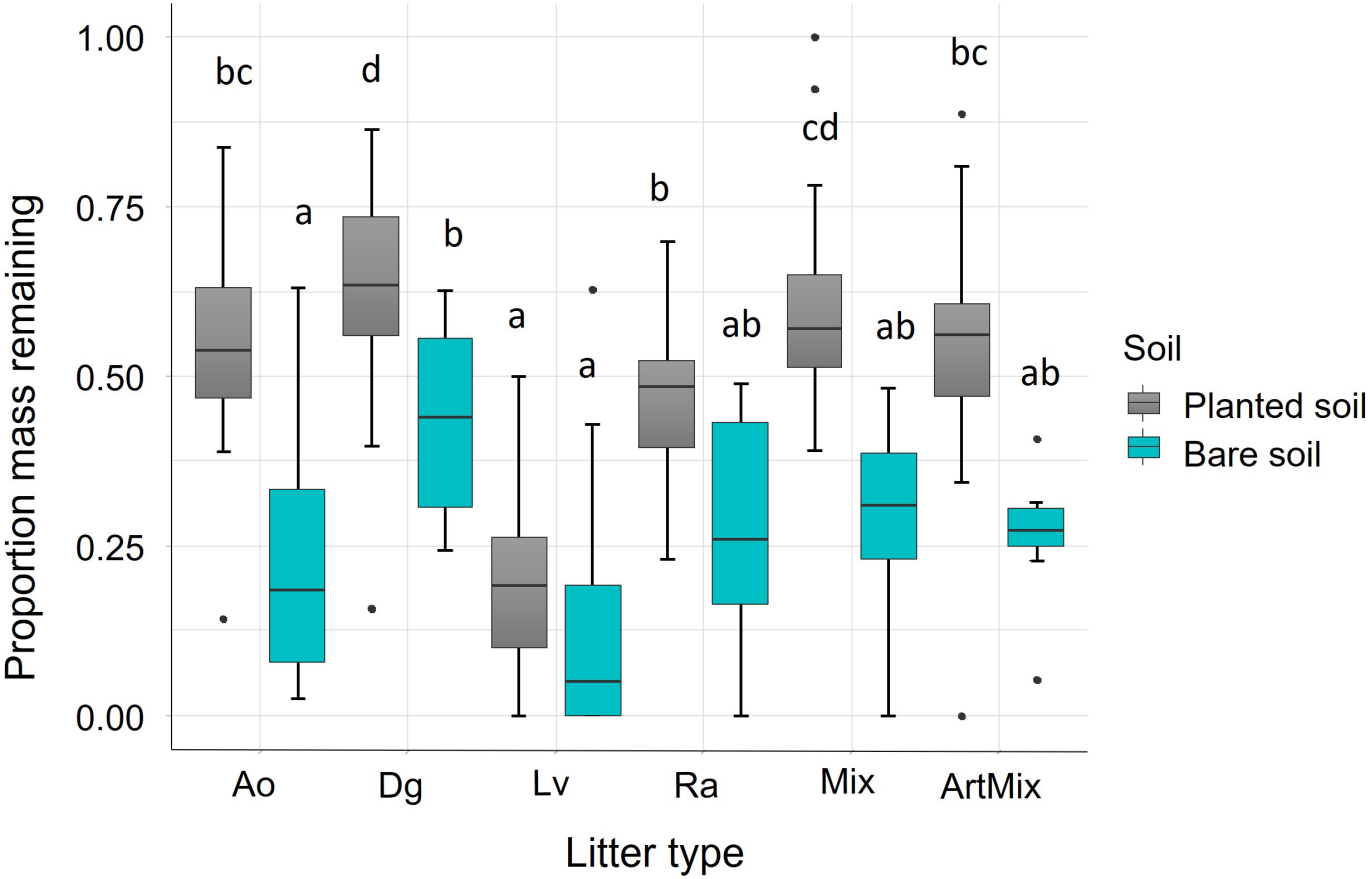
Mean remaining mass of each litter type in planted (grey) and bare soil (blue). Different letters indicate a statistically significant difference between groups (*p* < 0.05, Tukey HSD). Boxplots include litter decomposition data from two time points pooled together (central lines are medians, box top and bottom are first and third quartiles, whiskers are 1.5 times the interquartile range, black circles are outliers). Ao, *Anthoxanthum odoratum*; ArtMix, artificial mixture; Dg, *Dactylis glomerata*; Lv, *Leucanthemum vulgare*; Mix, natural mixture; Ra, *Rumex acetosa*.

There was a significant interaction between litter type and plant presence (*F*_5,129_ = 4.9, *p* < 0.001) where the negative effect of plant presence on root litter decomposition varied across litter species (Figure 4). Decomposition of *L. vulgare*, *R. acetosa* and the ‘artificial’ mixture litter was not affected by the presence of live plants, while decomposition of all other litter types was significantly reduced in the presence of living plants (Figure 4). The differences in decomposition rates between litter types were more pronounced when litter was incubated in planted communities (marginal *R* ^2^ = 0.64) than in bare soil (marginal *R* ^2^ = 0.33; Figure 4).

### 3.4 Home‐field advantage effects on litter decomposition

**FIGURE 5:**
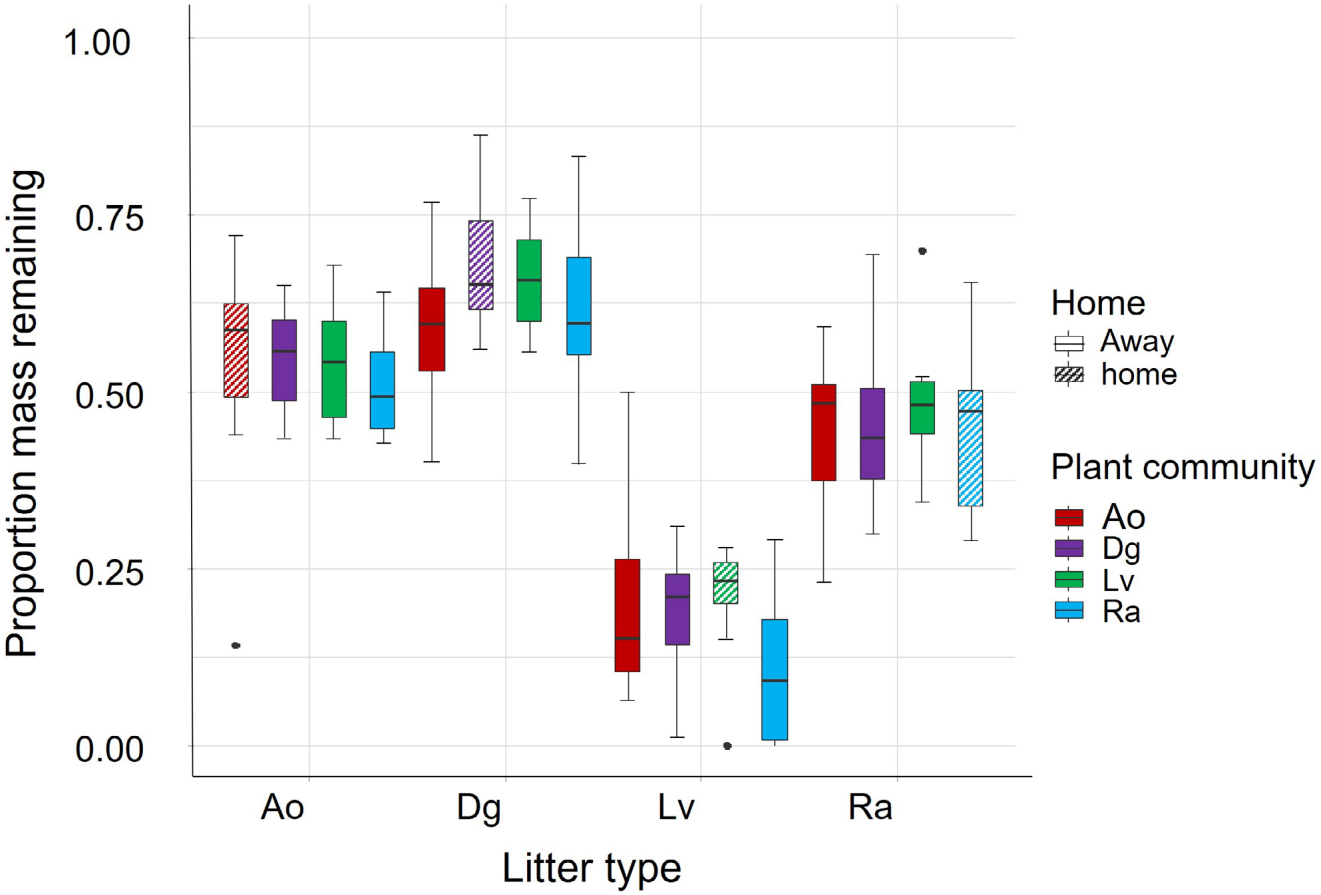
Decomposition of root litter when placed in its home (same plant community; hatched fill) and away (different plant community; solid fill) plant community. Boxplots represent the two litter decomposition data points pooled together (central lines are medians, box top and bottom are first and third quartiles, whiskers are 1.5 times the interquartile range, black circles are outliers). There was no statistically significant difference in litter decomposition between home and away (*p* > 0.05). Ao, *Anthoxanthum odoratum*; Dg, *Dactylis glomerata*; Lv, *Leucanthemum vulgare*; Ra, *Rumex acetosa*.

No significant home‐field advantage was detected for any litter type. Litter decomposition did not significantly differ when litters were placed in their ‘home’ (living plants belonging to the same species as the litter) compared with when placed in ‘away’ plant community (living plants different to the litter identity; Figure 5; Table S2).

### 3.5 Litter chemical composition

Litter types differed in their C:N ratios (*F*_5,135_ = 7.25, *p* < 0.001), with *L. vulgare* having the highest C:N ratio and *D. glomerata* the lowest. The artificial and natural mixtures had similar C:N ratios (Figure S4a).

### 3.6 Effects of litter and plant community identity on soil nutrient availability, microbial biomass and composition

Soil nutrient availability and microbial communities differed significantly between plant community treatments (Table S1). NH_4_^+^‐N concentrations differed between soils occupied by different plant species (Figure S4b, *F*_5,108_ = 5.28, *p* < 0.01), while NO_3_^−^‐N and total nitrogen concentrations were higher in bare soil than in all planted treatments (*F*_5,108_ = 66.58, *p* < 0.01, *F*_5,108_ = 25.54, *p* < 0.01, respectively; Figure S4c,d). There were no significant differences between treatments in dissolved organic carbon concentrations (Figure S4e; Table S3). Microbial biomass C was highest in bare soil, and monocultures of *L. vulgare* had the lowest microbial biomass C (*F*_5,108_ = 4.54, *p* < 0.01, Figure S4f). Total PLFA, bacterial PLFA, fungal PLFA, and the F/B ratio did not differ across treatments (Table S4).

## 4 DISCUSSION

We set out to test three unresolved hypotheses on the decomposition of root litter. First, we hypothesised that the decomposition of mixed root litter would be faster than predicted from the decomposition of the individual, single‐species litters because of complementarity effects in the consumption of mixed litter by decomposers. However, we found the opposite, with root litter produced in species mixtures and artificially mixed litter both decomposing at a slower rate than expected. Second, we expected natural mixtures (i.e. mixed root litter from plants that grew together) to decompose faster than ‘artificial’ mixtures (i.e. mixed root litter from plants that grew apart) because interspecific root interactions have been shown to increase litter quality. Instead, we found no difference between the decomposition of natural and artificial litter mixtures. Finally, we expected root litter to decompose faster in planted soil compared to bare soil because of a positive rhizosphere priming effect. In addition, we expected enhanced decomposition in the presence of living conspecific plants due to home‐field advantage effects. In contrast to this expectation, we found a clear negative priming effect on litter decomposition (i.e. root litter decomposed fastest in bare soil) and no home‐field advantage for any of the root litter types.

### 4.1 Slower decomposition of mixed‐species than single‐species litter

We found clear differences in decomposition rates between roots of individual species. We also detected significantly slower decomposition of litter produced in species mixtures compared to mean decomposition recorded for monoculture litters. Slower decomposition of litter produced in species mixtures may be due to changes in (i) root litter quality due to interspecific root‐root interactions, (ii) relative abundances of species with different litter quality in mixed communities and (iii) chemical interactions between fast and slow decomposing litters.

We found that litter mixtures of roots that had grown together (natural mixtures) did not decompose faster than artificial litter mixtures of roots that had never interacted. This finding suggests that interactions between roots of different species growing together did not affect root litter quality. This is in contrast with the limited evidence showing that intraspecific and interspecific interactions can induce changes in plant litter quality (Genung et al., 2013; Lak et al., 2020; Semchenko et al., 2017). For instance, it has been shown that root N concentrations under interspecific competition were lower than under intraspecific competition and single plants (Lak et al., 2020). Also, more genetically diverse mixtures of the same species produced root litter with higher N content (Semchenko et al., 2017).

In addition to changes in litter quality, interspecific root interactions may also affect the relative species abundances in mixed communities. Such changes in species relative abundance, particularly proportions of different growth forms with contrasting litter properties and decomposability, may be one of the main drivers of changes in litter decomposition along species richness gradients (Chen et al., 2017; Wang et al., 2022). However, we found that the decomposition of artificial root litter mixtures of known composition was still significantly slower than expected based on the decomposition rates of component litters in monoculture conditions. These findings suggest that slower decomposition in species mixtures was not driven by biotic interactions between plant species or changes in species relative abundances.

Slower decomposition of mixed litter may be due to chemical interactions between litters belonging to different species. While the addition of nutrient‐rich litter can enhance the decomposition of other litters by stimulating decomposer communities (Chapman et al., 1988; Hättenschwiler & Gasser, 2005), the presence of litter with high N content can also lead to the abiotic production of recalcitrant compounds. This may happen via the addition of amino groups onto phenols or quinones leading to their autopolymerisation (e.g. phenazine; Knicker, 2004). This process can make litter more resistant to microbial decomposition, inhibiting the oxidative enzymes that target lignin degradation and accumulating microbial residues resistant to decay (Hobbie, 2015). Similarly, secondary compounds (e.g. tannins) are known to inhibit decomposition by forming insoluble complexes with other polymers and inhibiting enzyme activities (Chomel et al., 2016). Chemical analysis of litter decomposition dynamics in mixed communities is necessary to test if such interactions can also contribute to reduced decomposition of litter mixtures (Dong et al., 2020; Guo et al., 2021). In addition, further investigations are needed to test the wider applicability of our findings, as this study included only four common grassland species. The inclusion of a wider range of species diversity and litter traits can result in stronger effects of plant diversity on root litter decomposition and change the relative importance of biotic and abiotic interactions in driving decomposition.

### 4.2 Negative priming of root litter decomposition

We found that root litter decomposition was faster in bare soil than in planted soil, which, while in contrast with our expectation, is in line with other studies (Barel et al., 2019; Cheng et al., 2014; Saar et al., 2016). This negative priming effect can be explained by preferential substrate utilisation and competition for N (Coq et al., 2011; van der Krift et al., 2002). Microbial biomass C and soil nitrate concentrations were highest in the bare soil treatment, indicating that living plants could have likely outcompeted microbes for nutrients and decreased microbial growth (Wardle et al., 2002). In addition, root exudates can act as a source of nutrients and carbon for microbial communities and induce a shift in microbial substrate preference from macromolecular C towards low molecular weight compounds (Shi et al., 2018). This mechanism may explain the slower litter decomposition in planted soil if root exudates shifted microbial community composition towards specialisation on root exudate consumption, rather than litter decomposition (Beidler et al., 2021; Saar et al., 2016). Supporting this mechanism, Nuccio et al. (2021) found clear niche differentiation in microbial communities depending on the substrate added, with specific microbes specialising on root litter and root exudates. Interestingly, the extent of priming effect varied significantly with litter identity, with *L. vulgare* litter showing the weakest priming effect but also the fastest decomposition rates with and without living roots. This suggests that the presence of living roots can steer microbial communities away from litter decomposition, particularly when there is a contrast between labile carbon in exudates and decomposability of litter. Also, litters belonging to different species had similar decomposition rates in bare soil. However, interspecific differences in decomposition rates became larger in the presence of living roots. Thus, living roots can importantly modify litter decomposition and interspecific differences in decomposition rates can be overlooked in litter incubation studies that do not include living plants.

### 4.3 The lack of home‐field advantage

The decomposition of root litter did not depend on whether the litter was incubated in the presence of its own ‘home’ species or a different ‘away’ species. Thus, we did not find evidence for a home‐field advantage in our experiment. The evidence for home‐field advantage is mixed, with some studies reporting positive effects (Ayres et al., 2009; Gholz et al., 2000; Vivanco & Austin, 2008), and other studies showing weak (Zheng et al., 2023) or non‐existent effects (Huangfu et al., 2019; Osburn et al., 2022). In greenhouse experiments, these differences in observed effects are mainly attributed to the soil disturbance that breaks down plant‐decomposer community interactions and excludes soil fauna (Veen et al., 2015). Some studies may also be too short for a specialised microbial community to develop (Ayres et al., 2009; Veen et al., 2015). The latter may be the main reason why we did not find a home‐field advantage. Our experiment only lasted 24 weeks, and no significant divergence in soil microbial communities based on PLFAs between different plant communities was detected. This is in line with similar studies (Cong et al., 2015; Coq et al., 2011).

## 5 CONCLUSIONS

By simultaneously manipulating root litter composition and the presence and composition of living plant communities, we identified litter composition and the presence, but not identity, of living roots as the major factors modifying root decomposition. We found that litter produced by mixed‐species communities decomposed significantly slower than monoculture litter. By quantifying the relative abundances of different species in root litter and assessing the decomposition of artificial root litter mixtures, we were able to exclude the effects of plant–plant interactions on species relative abundances and litter quality as mechanisms mediating the negative effect of litter mixing on decomposition. Finally, we found no evidence for home‐field advantage. This suggests that specialised decomposer communities did not play a significant role in our study system. However, we found a strong negative priming effect, which highlights the importance of interactions between microbial communities associated with living roots and root litter in modifying decomposition rates. Together, these findings increase our understanding of the controls on root litter decomposition and suggest that chemical interactions between substrates originating from litter of different species may be an important but poorly understood mechanism behind the negative effect of species richness on litter decomposition.

## AUTHOR CONTRIBUTIONS

All authors contributed equally toward the conceptualisation of the study. Cristina Heredia‐Acuña carried out greenhouse work and laboratory analysis. Cristina Heredia‐Acuña and Marina Semchenko analysed the data. Cristina Heredia‐Acuña prepared the first draft of the manuscript. Franciska T. De Vries and Marina Semchenko contributed to preparing the final version of the manuscript.

## CONFLICT OF INTEREST STATEMENT

The authors declare no conflict of interest. Marina Semchenko and Franciska de Vries are Associate Editors at *Journal of Ecology* but took no part in the peer review or decision‐making process for this manuscript.

### PEER REVIEW

The peer review history for this article is available at https://www.webofscience.com/api/gateway/wos/peer‐review/10.1111/1365‐2745.14207.

## Supporting information


**Figure S1:** Reference curves of estimated abundance (*y*‐axis) against actual species abundance in a sample (*x*‐axis) for all four plant species in the mixed root samples used for estimating species proportions after qPCR analysis. Each figure represents a different plant species. Plots are combinations of 24 ratio samples (0:1:3:6) and six standards (1:1:1:1). Blue lines represent linear regressions. Linear relationships were used to calculate grams of fresh weight of roots in mixed root litter.
**Figure S2:** Expected decomposition based on decomposition of monoculture litters against observed decomposition rates of natural root litter mixtures. There was a statistically significant difference between expected and observed decomposition (*p* < 0.05).
**Figure S3:** Correlations between the proportions of each plant species in natural litter mixtures (estimated using qPCR) and the difference between observed and expected proportions of litter mass remaining. Positive values indicated more than expected litter mass remaining, that is slower decomposition of litter mixtures than expected based on the decomposition of monoculture litter. No significant correlations were detected (*p* > 0.05). Ao, *Anthoxanthum odoratum*; Dg, *Dactylis glomerata*; Lv, *Leucanthemum vulgare*; Ra, *Rumex acetosa*.
**Figure S4:** (a) Mean of C:N ratios of all litter types; dissolved soil nitrogen pools (b–d), dissolved organic carbon (e) and microbial biomass carbon (f), in each plant community and in bare soil and. Different letters indicate a statistically significant difference between groups (*p* < 0.05, Tukey HSD). Boxplots central lines are medians, box top and bottom are first and third quartiles, whiskers are 1.5 times the interquartile range, blue triangles are means per treatment. Ao, *Anthoxanthum odoratum*; Dg, *Dactylis glomerata*; Lv, *Leucanthemum vulgare*; Ra, *Rumex acetosa*; Mix, Natural Mixture; AM, Artificial mixture; Bare, bare soil.
**Table S1:** The comparison of decomposition of root litter collected from species monocultures versus species mixtures, and natural species mixtures versus artificially mixed litter, when placed in different plant communities. Plant community type—species monoculture or mixture; plant community—plant community composition (unplanted, four monocultures and species mixture). Statistically significant effects (*p* < 0.05) are indicated in bold. Df, degrees of freedom; Df.res, residual degrees of freedom.
**Table S2:** Home field advantage on root litter decomposition across two sampling points. Statistically significant effects (*p* < 0.05) are indicated in bold. Df, degrees of freedom; Df.res, residual degrees of freedom.
**Table S3:** The effects of litter type and plant community, and their interactions, on soil nutrients and microbial biomass. Statistically significant effects (*p* < 0.05) are shown in bold. Df, degrees of freedom; Df.res, residual degrees of freedom.
**Table S4:** The effects of litter type and plant community on soil PLFAs. Statistically significant effects (*p* < 0.05) are indicated in bold. Df, degrees of freedom; Df.res, residual degrees of freedom.

## Data Availability

All data are available in Dryad Digital Repository https://doi.org/10.5061/dryad.6m905qg5r (Heredia‐Acuña et al., 2023).
