# Supplementary material for: Root litter decomposition is suppressed in species mixtures and in the presence of living roots
Source: J Ecol. 2023 Oct 13;111(11):2519–31. doi: 10.1111/1365-2745.14207 (PMC10976660; doi:10.1111/1365-2745.14207)
Supplement: Supplementary file 1 — Figure S1: Reference curves of estimated abundance (y‐axis) against actual species abundance in a sample (x‐axis) for all four plant species in the mixed root samples used for estimating species proportions after qPCR analysis. Each figure represents a different plant species. Plots are combinations of 24 ratio samples (0:1:3:6) and six standards (1:1:1:1). Blue lines represent linear regressions. Linear relationships were used to calculate grams of fresh weight of roots in mixed root litter. Figure S2: Expected decomposition based on decomposition of monoculture litters against observed decomposition rates of natural root litter mixtures. There was a statistically significant difference between expected and observed decomposition (p < 0.05). Figure S3: Correlations between the proportions of each plant species in natural litter mixtures (estimated using qPCR) and the difference between observed and expected proportions of litter mass remaining. Positive values indicated more than expected litter mass remaining, that is slower decomposition of litter mixtures than expected based on the decomposition of monoculture litter. No significant correlations were detected (p > 0.05). Ao, Anthoxanthum odoratum; Dg, Dactylis glomerata; Lv, Leucanthemum vulgare; Ra, Rumex acetosa. Figure S4: (a) Mean of C:N ratios of all litter types; dissolved soil nitrogen pools (b–d), dissolved organic carbon (e) and microbial biomass carbon (f), in each plant community and in bare soil and. Different letters indicate a statistically significant difference between groups (p < 0.05, Tukey HSD). Boxplots central lines are medians, box top and bottom are first and third quartiles, whiskers are 1.5 times the interquartile range, blue triangles are means per treatment. Ao, Anthoxanthum odoratum; Dg, Dactylis glomerata; Lv, Leucanthemum vulgare; Ra, Rumex acetosa; Mix, Natural Mixture; AM, Artificial mixture; Bare, bare soil. Table S1: The comparison of decomposition of root litter collected from [file JEC-111-2519-s001.docx]

**Supplementary Material from:**

**Root litter decomposition is suppressed in species mixtures and in the presence of living roots**

Authors:

**Cristina Heredia-Acuna**

Address: Royal College of Surgeons in Ireland, Department of Microbiology, 123 St Stephen's Green, Dublin 2, Ireland

Department of Earth and Environmental Sciences, University of Manchester, Oxford Road, Manchester, M13 9PL, UK

Email address: herediaac@gmail.com

**Marina Semchenko**

Address: Institute of Ecology and Earth Sciences, University of Tartu, Liivi 2, 50409, Tartu, Estonia

Department of Earth and Environmental Sciences, University of Manchester, Oxford Road, Manchester, M13 9PL, UK

Email Address: marina.semchenko@ut.ee

**Franciska T. De Vries**

Address: Institute for Biodiversity and Ecosystem Dynamics, University of Amsterdam, 1090 GE Amsterdam, The Netherlands

Department of Earth and Environmental Sciences, University of Manchester, Oxford Road, Manchester, M13 9PL, UK

Email address: f.t.devries@uva.nl

**Supplementary Material**

**
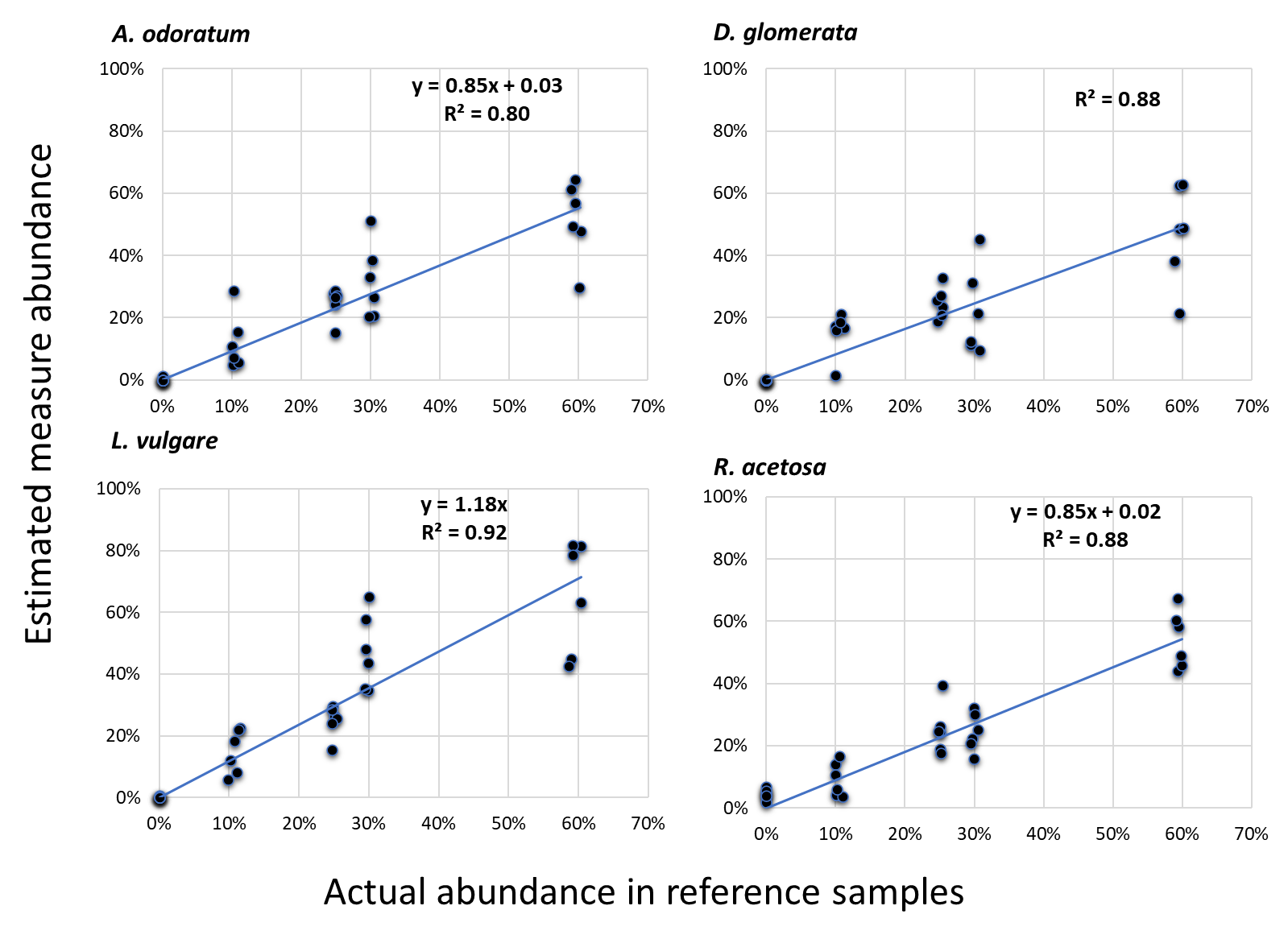
**

**Suppl. Figure 1.** Reference curves of estimated abundance (y-axis) against actual species abundance in a sample (x-axis) for all four plant species in the mixed root samples used for estimating species proportions after qPCR analysis. Each figure represents a different plant species. Plots are combinations of 24 ratio samples (0:1:3:6) and six standards (1:1:1:1). Blue lines represent linear regressions. Linear relationships were used to calculate grams of fresh weight of roots in mixed root litter.


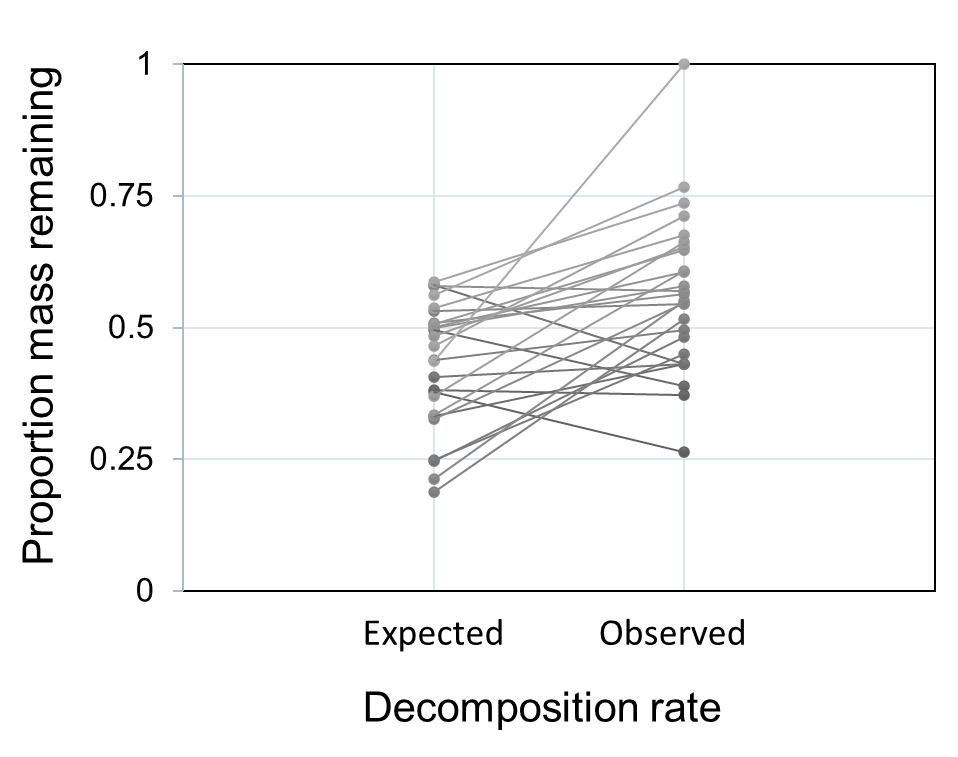


**Suppl. Figure 2.** Expected decomposition based on decomposition of monoculture litters against observed decomposition rates of natural root litter mixtures. There was a statistically significant difference between expected and observed decomposition (*P* < 0.05).


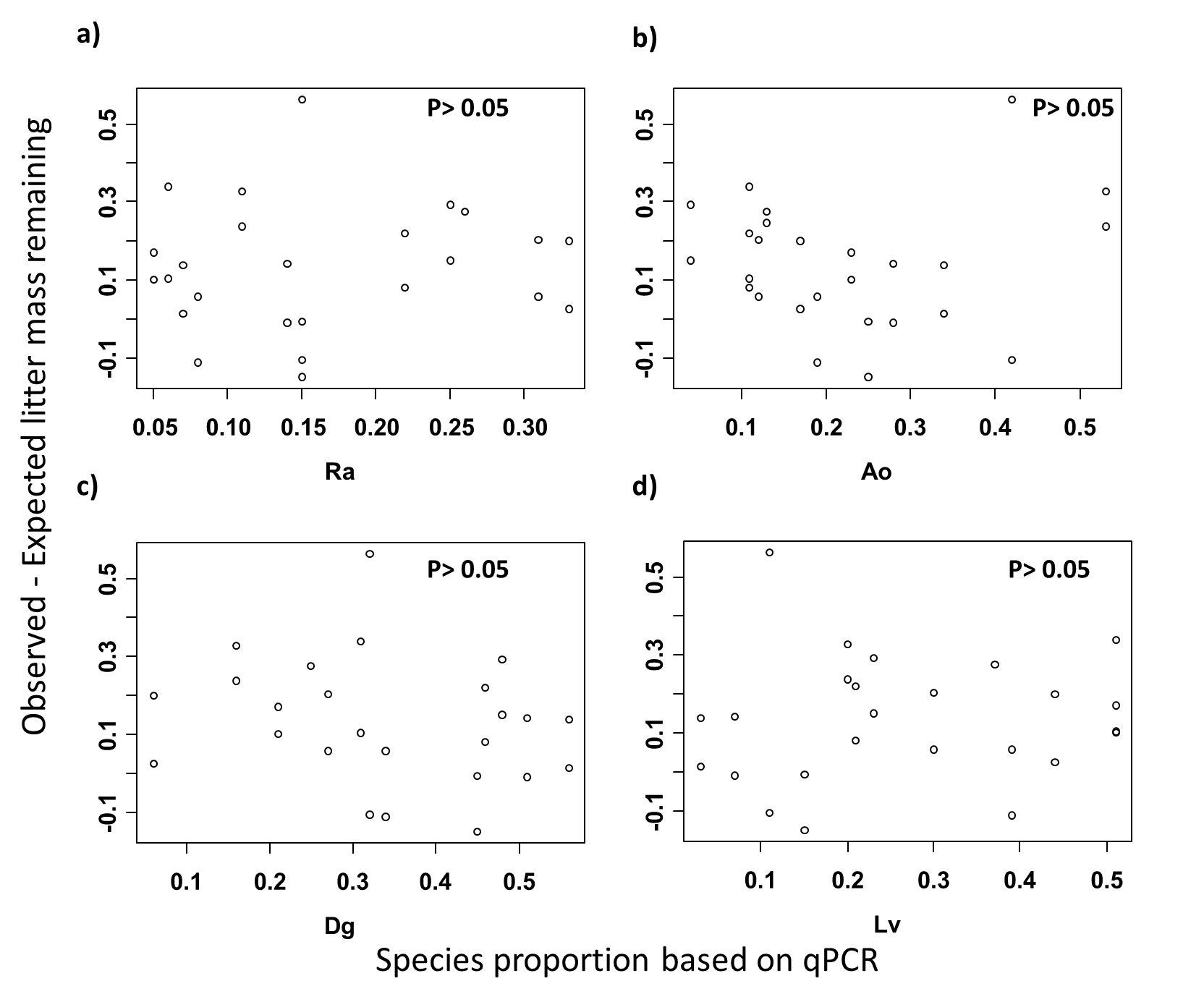


**Suppl. Figure 3.** Correlations between the proportions of each plant species in natural litter mixtures (estimated using qPCR) and the difference between observed and expected proportions of litter mass remaining. Positive values indicated more than expected litter mass remaining, i.e., slower decomposition of litter mixtures than expected based on the decomposition of monoculture litter. No significant correlations were detected (P > 0.05). Ao= *Anthoxanthum odoratum*, Dg= *Dactylis glomerata*, Lv= *Leucanthemum vulgare*, Ra= *Rumex acetosa*.

**
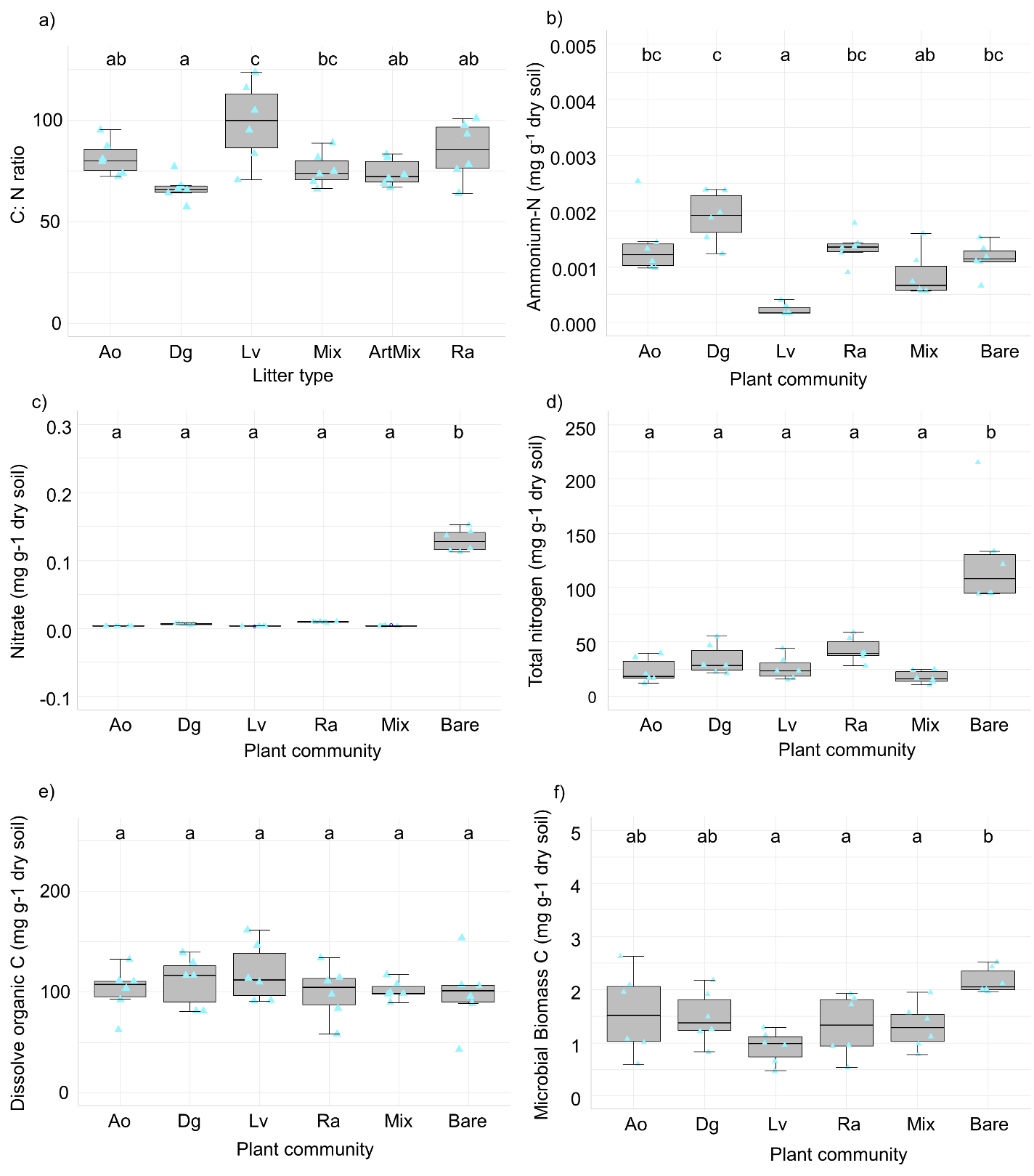
Suppl. Figure 4.** a) Mean of C:N ratios of all litter types; dissolved soil nitrogen pools (b-d), dissolved organic carbon (e) and microbial biomass carbon (f), in each plant community and in bare soil. Different letters indicate a statistically significant difference between groups (*P* < 0.05, Tukey HSD). Boxplots central lines are medians, box top and bottom are first and third quartiles, whiskers are 1.5 times the interquartile range, blue triangles are means per treatment. Abbreviations: Ao= *Anthoxanthum odoratum*, Dg*= Dactylis glomerata*, Lv= *Leucanthemum vulgare*, Ra= *Rumex acetosa*, Mix=Natural Mixture, ArtMix = Artificial mixture, Bare= bare soil

**Table S1.** The comparison of decomposition of root litter collected from species monocultures *versus* species mixtures, and natural species mixtures *versus* artificially mixed litter, when placed in different plant communities. Plant community type - species monoculture or mixture; plant community – plant community composition (unplanted, four monocultures and species mixture). Statistically significant effects (*P* < 0.05) are indicated in bold. Df = degrees of freedom, Df.res = residual degrees of freedom.

| Analysis | Source of variation | F | Df | Df.res | ***P*** |
| --- | --- | --- | --- | --- | --- |
| ***Monoculture litter vs natural litter mixture*** | | | | | |
|  | Litter type | 9.65 | 1 | 93 | **<0.01** |
|  | Plant community type | 0.53 | 1 | 93 | 0.47 |
|  | Time | 28.51 | 1 | 96 | **<0.01** |
|  | Litter type: Plant community type | 0.0004 | 1 | 93 | 0.98 |
|  | Litter type: Time | 0.19 | 1 | 96 | 0.66 |
|  | Plant community type: Time | 0.48 | 1 | 96 | 0.49 |
|  | Litter type: Plant community type: Time | 0.25 | 1 | 96 | 0.61 |
|  |  |  |  |  |  |
| ***Natural litter mixture vs artificial litter mixture*** | | | | | |
|  | Litter type | 2.62 | 1 | 33 | 0.11 |
|  | Plant community | 14.60 | 5 | 33 | **<0.01** |
|  | Time | 7.18 | 1 | 36 | **0.01** |
|  | Litter type: Plant community | 0.65 | 5 | 33 | 0.66 |
|  | Litter type: Time | 0.72 | 1 | 36 | 0.39 |
|  | Plant community: Time | 1.79 | 5 | 36 | 0.14 |
|  | Litter type: Plant community: Time | 0.51 | 5 | 36 | 0.77 |

**Table S2.** Home field advantage on root litter decomposition across two sampling points. Statistically significant effects (*P* < 0.05) are indicated in bold. Df = degrees of freedom, Df.res = residual degrees of freedom.

| Analysis | Source of variation | F | Df | Df.res | ***P*** |
| --- | --- | --- | --- | --- | --- |
|  |  |  |  |  |  |
|  | Litter type | 129.23 | 3 | 53 | **<0.01** |
|  | Home away | 0.97 | 1 | 53 | 0.33 |
|  | Time | 35.24 | 1 | 56 | **<0.01** |
|  | Litter type: Home away | 0.74 | 3 | 53 | 0.53 |
|  | Litter type: Time | 1.01 | 3 | 56 | 0.39 |
|  | Home away: Time | 2 | 1 | 56 | 0.16 |
|  | Litter type: Home away: Time | 1.96 | 3 | 56 | 0.13 |

**Table S3.** The effects of litter type and plant community, and their interactions, on soil nutrients and microbial biomass. Statistically significant effects (*P* < 0.05) are shown in bold. Df = degrees of freedom, Df.res = residual degrees of freedom.

| Analysis | Source of variation | F | Df | Df.res | ***P*** |
| --- | --- | --- | --- | --- | --- |
| Total dissolved N | Litter type | 0.85 | 5 | 108 | 0.51 |
|  | Plant community | 25.54 | 5 | 108 | **<0.01** |
|  | Litter type: Plant community | 1.27 | 25 | 108 | 0.22 |
| Ammonium-N | Litter type | 0.20 | 5 | 108 | 0.96 |
|  | Plant community | 5.28 | 5 | 108 | **<0.01** |
|  | Litter type: Plant community | 0.45 | 25 | 108 | 0.98 |
| Nitrate | Litter type | 0.17 | 5 | 108 | 0.97 |
|  | Plant community | 66.58 | 5 | 108 | **<0.01** |
|  | Litter type: Plant community | 0.21 | 25 | 108 | 1.000 |
| Dissolved organic C | Litter type | 0.25 | 5 | 108 | 0.93 |
|  | Plant community | 0.20 | 5 | 108 | 0.96 |
|  | Litter type: Plant community | 0.36 | 25 | 108 | 0.99 |
| Microbial biomass (C) | Litter type | 0.28 | 5 | 108 | 0.92 |
|  | Plant community | 4.54 | 5 | 108 | **<0.01** |
|  | Litter type: Plant community | 1.31 | 25 | 108 | 0.17 |

**Table S4**. The effects of litter type and plant community on soil PLFAs. Statistically significant effects (*P* < 0.05) are indicated in bold. Df = degrees of freedom, Df.res = residual degrees of freedom.

| Analysis | Source of variation | F | Df | Df.res | ***P*** |
| --- | --- | --- | --- | --- | --- |
| ***Total PLFAs*** |  |  |  |  |  |
|  | Litter type | 0.63 | 5 | 108 | 0.67 |
|  | Plant community | 0.24 | 5 | 108 | 0.94 |
|  | Litter type: Plant community | 0.59 | 25 | 108 | 0.93 |
| ***Total fungi*** |  |  |  |  |  |
|  | Litter type | 0.62 | 5 | 108 | 0.68 |
|  | Plant community | 0.30 | 5 | 108 | 0.91 |
|  | Litter type: Plant community | 0.56 | 25 | 108 | 0.94 |
| ***Total bacteria*** |  |  |  |  |  |
|  | Litter type | 0.61 | 5 | 108 | 0.68 |
|  | Plant community | 0.24 | 5 | 108 | 0.94 |
|  | Litter type: Plant community | 0.58 | 25 | 108 | 0.93 |
| ***Bacteria Gram positive*** | |  |  |  |  |
|  | Litter type | 0.62 | 5 | 108 | 0.68 |
|  | Plant community | 0.16 | 5 | 108 | 0.97 |
|  | Litter type: Plant community | 0.61 | 25 | 108 | 0.91 |
| ***Bacteria Gram negative*** | |  |  |  |  |
|  | Litter type | 0.61 | 5 | 108 | 0.69 |
|  | Plant community | 0.29 | 5 | 108 | 0.91 |
|  | Litter type: Plant community | 0.57 | 25 | 108 | 0.94 |
| ***Fungal: Bacteria ratio*** | |  |  |  |  |
|  | Litter type | 0.55 | 5 | 108 | 0.73 |
|  | Plant community | 0.46 | 5 | 108 | 0.80 |
|  | Litter type: Plant community | 1.50 | 25 | 108 | 0.08 |
